# Supplementary material for: Freeform 3D Bioprinting Involving Ink Gelation by Cascade Reaction of Oxidase and Peroxidase: A Feasibility Study Using Hyaluronic Acid-Based Ink
Source: Biomolecules. 2021 Dec 20;11(12):1908. doi: 10.3390/biom11121908 (PMC8699251; doi:10.3390/biom11121908)
Supplement: Supplementary file 1 [file biomolecules-11-01908-s001.zip › biomolecules-1484944-supplementary.pdf]

Supplementary Materials

Freeform 3D bioprinting involving ink gelation by cascade reaction of oxidase and peroxidase: A feasibility study using hyaluronic acid-based ink

Shinji Sakai*, Ryohei Harada, Takashi Kotani

Division of Chemical Engineering, Department of Materials Engineering Science, Graduate School of Engineering Science, Osaka University, 1-3 Machikaneyama-cho, Toyonaka, Osaka 560-8531, Japan.
*E-mail: sakai@cheng.es.osaka-u.ac.jp

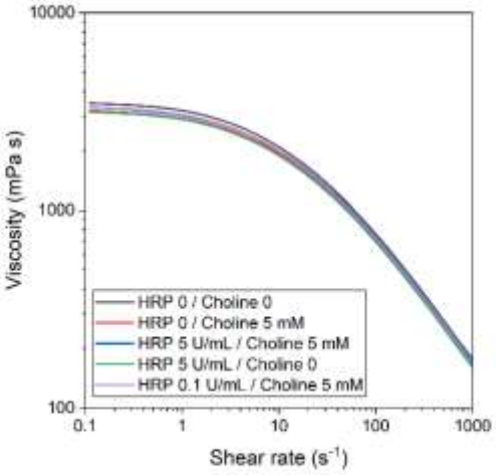

Figure S1. Effect of the contents of HRP and choline on the shear rate-viscosity properties of inks containing 1.25% (*w*/*v*) hyaluronic acid derivative possessing phenolic hydroxyl moieties. The shear rate-viscosity properties were measured using a rheometer (HAAKE MARS III, Thermo Fisher Scientific, Walthan, MA) at 25 °C.

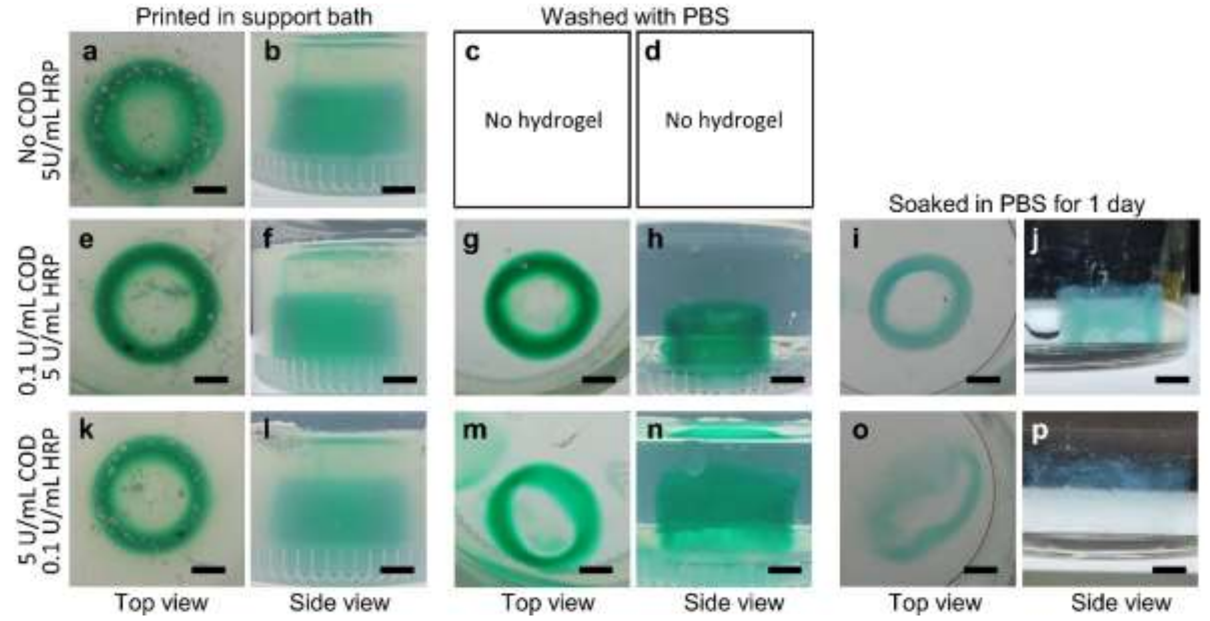

Figure S2. Effect of the contents of COD in support bath and HRP in ink on the printing of cylindrical-shaped hydrogel constructs. **a, b, e, f, k, l**) just after extruding ink containing 1.25% (*w*/*v*) hyaluronic acid derivative possessing phenolic hydroxyl moieties, 5 mM choline chloride, and HRP into support baths containing 1.0% (*w*/*v*) xanthan gum and COD, and **c, d, g, h, m, n**) after washed with PBS for removing xanthan gum, and **i, j, o, p**) the constructs after 1 day of soaking in PBS. The contents of COD and HRP are shown on the left side of panels **a, e, k**. Bars: 5 mm.
